# The neighborhood effects on the online financial investment of rural households: Evidence from China

**DOI:** 10.1371/journal.pone.0296972

**Published:** 2024-03-29

**Authors:** Wenxian Li, Kefei Han, Zhenyu Lao, Yuyuan Chen

**Affiliations:** 1 School of Finance, Southwestern University of Finance and Economics, Chengdu, 611130, China; 2 School of Finance, Tianfu College of SWUFE, Mianyang, 621050, China; University of Almeria: Universidad de Almeria, SPAIN

## Abstract

Neighborhood effects are a common strategy for rural households to deal with irrational situations such as deficient information and ability. Based on the 2019 CHFS survey data, we designed a Probit model to verify whether neighborhood effects exist in the online financial investment of rural households. Our paper constructs a multiple mediation model to explore its mechanism. Otherwise, we execute the heterogeneity analysis by dividing the total sample into groups. Our paper proved that (1) Rural households have significant neighborhood effects on online financial investment. (2) Heterogeneity analysis shows that neighborhood effects are stronger among women, the younger, low-education, and low-income rural households. (3) Through the multiple mediation model, we proved that the neighborhood effects on online financial investment of the peasant household work by the financial knowledge spillover and risk-taking enhancement. Our study conduces to a better understanding of the financial decision-making of rural households, which may provide a practical implication for the popularization of new financial products and the optimal design of policy interventions.

## 1. Introduction

China’s economy has shown rapid development in recent decades, and so has a wealth of urban and rural residents. According to the Statistical Communique on National Economic and Social Development of the People’s Republic of China (2022), rural residents’ per capita disposable income was 20,133 CNY (China Yuan) by 2022, leading to a constant increase in financial needs. However, the efficiency of rural household asset allocation is low. Wealth management is a tool for rural households to smooth consumption and deal with risk, which is good for improving the welfare of rural families. In comparison, the current wealth management situation is not conducive to the improvement of the welfare of rural households.

In China, only 6.8% of peasants have bought stocks, 5.2% have bought fund products, and up to 55% are unsatisfied with their asset allocation. The two most important reasons lie in the lack of investment approaches and financial knowledge deficit, accounting for 35.76% and 72.22%, respectively. On the one hand, the financial products of traditional financial institutions have a high capital threshold and complicated operation process, which do not match the asset features and investment ability of peasant households. Besides, most peasants with poor financial literacy reject financial products due to vague aversion and risk aversion.

Online financial products refer to those launched by online financial platforms in China, including Yu’ebao, WeChat, Baidu Baiearn, etc. Financial products issued by banks, securities companies, fund companies, trust companies, and other financial institutions are excluded. Compared with traditional financial products, online financial investment has apparent technical advantages, effectively reducing the capital threshold restriction and transaction costs of financial planning. Therefore, they can meet the financial needs of rural households well in theory. However, online financial investment does not play the expected role in the wealth management of rural households in practice. Only 11.23% of rural households invest in online financial products. The main reason why they did not purchase online financial investment products, accounting for 72.10%, was that they did not know enough. Therefore, to improve their wealth management status and welfare, it is necessary to determine the factors influencing the purchase.

Mainstream economics standardizes individual financial decision-making as a process of rationally weighing risks, gains, and losses—driven by maximum utility. However, peasant households are usually limited in information and financial knowledge. They tend to make the trade-off between profits and losses through convenient methods with limited rationality, such as "empirical systems" and "heuristics." Similar decisions from neighbors will be a helpful offset for rural households to deal with the deficiency of information and financial knowledge through the "demonstration-imitation effect" and "social comparison." Besides, rural areas are primary villages based on kinship and marriage in China. Households in the same village face similar risks and information status with similar cultural backgrounds. And they attach importance to the consistency with the other villagers driven by collective solid consciousness. So, rural households’ economic decision-making will converge with the neighbors, especially when faced with high risk, asymmetric information, and other irrational states.

Above all, irrational characteristics and neighborhood effects should be considered when studying the financial behavior of rural households. Neighborhood effects is the phenomenon in which neighbors influence individuals’ behavior through interactions such as learning, imitating, and sharing. (Bursztyn et al., 2014; Bollinger et al., 2018; Li et al., 2021) [1–3]. Therefore, exploring neighborhood effects and how they work is essential to analyzing rural households’ financial decision-making and rural financial policy-making. Our paper offers a perspective of the ’neighborhood effects’ to explore the online financial investment of rural households. Based on the data from the 2019 CHFS (China Household Finance Survey and Research Center), we empirically verify the neighborhood effects on rural households’ online financial investment. Besides considering the differences in age, gender, education level, and income level of different individuals, our paper also uses heterogeneity analysis to identify the individuals whose neighborhood effects are more potent and which groups influence the others more significantly. Moreover, by utilizing a multiple mediation model in which financial knowledge and risk-taking are mediating variables, we detect the mechanism of how neighborhood effects work. Our study has the following contributions: (1) Verifying the existence of neighborhood effects in the decision-making process of rural households’ online financial investment to enrich the research in related fields. (2) Through heterogeneity analysis and mechanism analysis, helping further understand which individuals are more inclined to show neighborhood effect in their online financial investment decision-making process and the decision-making mechanism of rural households from the perspective of social interaction. (3) Providing a practical implication for popularizing new financial products and the optimal design of policy interventions in rural areas.

We organize the rest of the part as follows. Section 2 makes a literature review illustrating relevant research on neighborhood effects. Section 3 proposes three hypotheses. Section 4 introduces the data and empirical methodology. Section 5 presents the empirical results and appropriate tests. Section 6 is the conclusion and discussion, summarizing the research conclusions.

## 2. Literature review

Through strict assumptions and careful reasoning, financial economics makes it clear whether investors participate in financial markets: Investors are willing to purchase risky assets as long as their expected return is higher than the risk-free rate. However, households’ financial decision-making is not as predicted by classical theory—a significant fraction of households do not participate in risky financial markets or allocate risky assets. In reality, individuals are subject to multiple constraints. As a result, the economic behavior of individuals has significant heterogeneity, and factors affecting household financial behavior go beyond the risk and return characteristics of financial assets.

The characteristics of residents’ financial management and financial asset allocation are essential determinants of financial participation. Strong risk aversion, a single asset allocation structure, a predominance of passive preventive financial planning, and a high proportion of property investment characterize Chinese residents’ financial management (Wu et al., 2010; Li et al., 2013) [4,5]. Based on this characteristic, Chinese residents’ allocation of risk financial assets is insufficient, and the risk and return of financial management are not completely matched, which leads to low efficiency of asset allocation and low satisfaction of residents’ financial management. (Zhou et al., 2018) [6]. Under the circumstances, it is necessary to explore the determinants of residents’ asset allocation to improve the efficiency of family asset allocation.

Vast empirical studies have investigated the determinants of individual asset allocation. The share of equity holdings and risk-free assets in residents’ financial assets show a "U" shaped relationship with age(Sun et al., 2021) [7]. Income, total wealth, education level, and financial literacy can significantly promote the proportion of residents’ financial assets holding and motivation for risk investment(Sarantsev, 2021; Tsagkanos & Athanasios, 2017) [8,9]. Moreover, residents’ health status and neighborhood effects also affect their participation in the stock market (Crainich et al., 2016; Bianchi, 2018) [10,11]. In addition, family financing is inevitably affected by the macro-economic condition. Easy money policy leads to lower interest rates, followed by a change in household financial asset allocation (Guerello, 2017) [12]. However, underdeveloped financial markets and insufficient financial instruments can weaken this mechanism (Rejeb & Boughrara, 2013) [13]. In contrast, fiscal policies and social security impact more residents’ participation in the financial market (Li et al., 2021) [14]. Furthermore, how developed the financial market is playing a role in promoting residents’ participation in financial markets (Smimou, 2014) [15]. In contrast, less literature pays attention to the role of online financial products in asset allocation of peasant’s households.

The online financial products refer to those launched by online financial platforms in China, including Yu’ebao, WeChat, Baidu Baiearn, etc. Financial products issued by banks, securities companies, fund companies, trust companies, and other financial institutions are excluded. Derived from crowd-funding and ’make a little a mickle’ thinking, online financial products are flexible, approachable, and abundant, and supported by online technologies such as online dealing to effectively reduce the capital threshold and transaction cost of financial planning (Xia & Hou, 2016) [16]. Most studies of the investment decision-making process of online financial products were carried out from the perspectives of behavioral attitude, perceived usefulness, perceived ease of use, and privacy risk (Benndorf et al., 2015; Nurul et al., 2016) [17,18]. Some also focus on the perceived enabling conditions, compatibility, subjective norms, and self-efficacy (Ithriah et al., 2020) [19]. A few researches are based on the combination of TPB (Planned Behavior Theory) and TAM (Technology Acceptance Model) (Ha et al., 2019) [20]. Additionally, other scholars proposed that convenience, performance expectation, effort expectation, perceived risk, trust tendency, and age affect online financial participation (Benndorf et al., 2015; Alnassar&Aloud, 2021) [17,21].

Existing research on online investment products focused more on market and individual factors, but ignore the social interaction among residents. Chinese residents’ online finance participants based on individual-bound rationality (Jin, 2015) [22], and individual’s financial decision-making process depends on individual subjective factors such as their own risk preference, knowledge level and wealth management objectives(Li & Mao,2010) [23]. However, they fail to be sensible due to their low financial literacy. Simultaneously, people can be affected by others from social networks and specific surroundings when making decisions (Frydman, 2015; Ouimet & Tate, 2020) [24,25]. Neighborhood effects are the phenomenon in which neighbors influence individuals’ behavior through interactions such as learning, imitating, and sharing (Bursztyn et al., 2014; Bollinger et al., 2018; Li et al., 2021) [1–3]. The communication among neighbors can be categorized as a case of neighborhood effects. It also is the result that residents passively receive information from others in the same village and follow suit consciousness based on group awareness (Sacerdote, 2001; Niu et al., 2020) [26,27].

Sociologists and educationalists proposed neighborhood effects firstly, which focused on the explanation of how others influence individual learning, non-cognitive ability, and preference (Lavy & Schlosser, 2011) [28]. A growing literature has paid close attention to neighborhood effects on decision situations. The literature on how neighbors may impact individuals’ decision-making processes stems from diverse disciplines, not economics and psychology. Scholars have extended the research on neighborhood effects to economics, management, finance, and other fields, including corporate innovation, financial arrangement, investment, and financing (Matray, 2014; Foucault & Fresard, 2014;Frydman, 2015) [29–31]. Moreover, the focus is also on individual gambling participation, entrepreneurial behavior, and donation behavior (Gamba et al., 2014; Monson et al., 2018; Drouvelis & Marx, 2020) [32–34]. In addition, the impact of neighborhood effects on individual wrong behaviors results in suggestions on negative behaviors, individual crime rates, and illegal decisions of companies through social multipliers (Kim, 2016; Berg & Nelson, 2016; Gerlinger & Hipp, 2020) [35–37].

A comprehensive analysis of existing research literature shows little literature on rural family Internet finance, and few scholars pay attention to the neighborhood effect on rural family Internet finance participation. So, our paper will explore the role of neighborhood effects in online financial products of peasant households and do further research on the mechanism.

## 3. Research hypothesis

Investing in online financial products is the decision-making process based on utility maximization, and rural households’ investment intention, investment knowledge, and expectations of profit and loss will be related to their neighbors. The research hypotheses are proposed based on the relevant theories and existing studies.

According to group reference and social comparison theory, a positive correlation exists between rural households’ online financial investment and the neighboring (Xu et al., 2017) [38]. On the one hand, the vague aversion to online financial investment results can be alleviated by the observed investment of neighbors with a similar background (Dimmock et al., 2016) [39]. On the other hand, the sense of belonging, recognition, and relative position in a specific group is noticeable to the members when making economic decisions (Goodrich & Hoij, 2014) [40]. Besides, rural residents are afraid to become ’special’ under the cultural background of the ’Doctrine of the Mean’ in China, so they are inclined to be in line with their neighbors. So, they tend to follow their neighbors in online investment decision-making.

H1: There are neighborhood effects in the online financial investment of rural households. Namely, the investment of rural households positively correlated with their neighbors’ investment.

Financial knowledge is a positive factor in an individual’s financial investment. The social learning theory indicates that communicating and sharing with their neighbors can expand the financial knowledge of rural residents (Lobel & Adler, 2015) [41]. Observing and imitating, individuals can learn how to invest online and estimate the gains and losses (Easaw & Mossay, 2015) [42]. As a result, peasant households will have similar knowledge about online financial investment with their neighbors, which leads to the same relevant decision. Further, the investment in online financial products from peasant households will be positively related to the similar behavior of others in the same village. Therefore, neighborhood effects of online investment would work by sharing and diffusing financial knowledge about online investment.

Besides, compared with urban residents, peasant households confront more information asymmetry. Though information channels have broadened with the rapid development of internet information technology, rural residents receive more excessive and fake information, which would confuse and mislead the unskilled and non-rational ones (Rana & Hokeen, 2018) [43]. Social networks such as relatives, neighbors, and colleagues deal with poor relevant information and knowledge (Acemoglu et al., 2011) [44]. Peasants effectively make up for the deficit and correct their irrational ideas in time by observing and communicating (Kawaguchi et al., 2019) [45], hence a broader scope of financial knowledge (Zhou et al., 2020) [46]. Subsequently, this alleviates the refusal of online financial investment caused by information deficiency and ambiguity aversion.

H2: Neighbors’ Online financial investments will enrich rural households’ financial knowledge through financial knowledge spillover, promoting their online financial investment. Namely, financial knowledge is one of the mechanisms of how the neighborhood effects work.

Online financial investment is a risk-taking process. Neighbors might influence individual risk preferences (Gortner & Weele, 2019) [47]. When a resident’s venture investment benefits, this sense of success is proportional to the number of investors in the same village. And the frustration of venture losses decreases with the number of investors around (Peake et al., 2013) [48]. On the one hand, the increasing number of online investors from neighbors will mitigate rural residents’ anxiety about risk-tolerating. On the other hand, neighbors’ decision-making can be a reference for rural households, enriching their information source and reducing risk ambiguity (Ahern et al., 2014) [49].

According to the emotional contagion theory, the willingness of rural families to take risks will be influenced and assimilated by neighbors when imitating and communicating. Specifically, rural residents with optimistic market predictions and less anxiety about risk-taking prefer more online investment. In this case, when observing more optimistic market predictions and less stress about risk-taking from their neighbor, rural residents would like to take more risks and invest more in online financial products (Joshy et al., 2015; Santini et al., 2015) [50,51].

H3: Neighborhood financial investments can increase rural households’ risk tolerance willingness and promote online financial investment. Namely, risk tolerance willingness is one of the mechanisms of how the neighborhood effects work.

**Fig 1:**
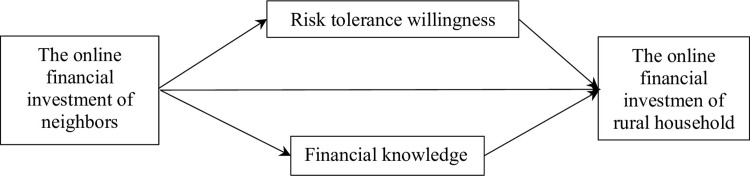
Research framework.

We have described the research framework in Fig 1 based on the three hypotheses above.

## 4. Data and empirical specification

### 4.1 Data description

The data were taken from CHFS (China Household Finance Survey) in 2019. It is a nationwide survey conducted by the China Household Finance Survey and Research Center of the Southwestern University of Finance and Economics (SWUFE). The proportional to population size (PPS) is adopted to select samples nationwide. The survey centers on data from 29 provinces and 345 counties, including 1,054 urban and rural communities. The demographic characteristics of CHFS, with a low rejection rate, are very close to that of the national census statistics. Therefore, the data is reliable and representative.

In the questionnaire, family (also individuals) and community are involved. First, we collect the basic demographic characteristics, assets and liabilities, income and consumption, insurance and security, employment, and subjective attitude of urban and rural families. The information above reflects the basic situation of families. On the other hand, the community questionnaire covers the basic situation of the community, politics, economy, culture, public security, environmental protection, etc.

Specifically, in household assets, there are more than business assets, houses, cars, and other non-financial assets in the questionnaire. We also inquire more details about the household’s financial assets, including cash, current deposit, time deposit, stocks, bonds, funds, financial products and derivatives, the renminbi assets, precious metals, loans, and other financial assets. In terms of household liabilities, we investigated the asset of each household (agriculture, business, housing, vehicles, non-financial assets, financial assets) and household liabilities. The information above supports our study on rural household investment in online products. Before analyzing, samples without key variables were removed, and the number of effective samples was finally 7165, thus the avoidance of the extreme values’ impact on the estimated results.

Table 1 shows the characteristics of samples. 48.09% of the questionnaire respondents are female. The respondents have an average age of 48.46, of which 3487 range from 46 to 60 years old, accounting for 34.71%. Nearly 40% of the respondents have 9 years of schooling or below, half of the respondents have attended a high school (9–12 years), and less than 10% of people have 13 years of schooling and beyond. About 20.52% of interviewees stated that their household annual income is less than 30,000 Yuan, and 8.68% of them have an annual income above 90,000 Yuan.

**Table 1:** Demographic profiles of the respondents.

	Category	Frequency	Proportion
Gender	Male	3719	51.91%
	Female	3446	48.09%
Age (years)	<25	396	5.52%
	25–35	825	11.51%
	36–45	2158	30.12%
	46–60	2487	34.71%
	>60	1299	18.12%
Schooling years	≤9 years	2518	35.14%
	10–12 years	4001	55.84%
	>12 years	646	9.02%
Household annual income (thousand yuan)	<30	1470	20.52%
	30–60	3888	54.27%
	60–90	1185	16.53%
	>90	622	8.68%

### 4.2 The identification of neighborhood effects

There are several challenges associated with identifying the neighborhood effects. Above all, it is difficult to define an appropriate’ neighbor group’ (Chen et al., 2010; Kim, 2016) [35,52]. Besides, the identification of neighborhood effects also faces three common concerns (Mansk,2013; Atefi&Pourmasoudi, 2019) [53,54]: (1) The contextual effect, which means that the behavior of an individual varies with the exogenous characteristics of the neighbors. (2) The correlated effect refers to the concern that households select neighbors according to their preferences and backgrounds (also called the ’self-selection problem’). For example, rural residents making similar online investment decisions may be because the local house prices attract people with similar income levels to live together, or they are affected by a common political factor. (3) Simultaneity, also termed a ’reflection problem,’ indicates that individuals and their peer-group influence each other simultaneously. There is a mutually causal relationship between personal behavior and neighbors’ behaviors, which would generate an endogenous issue when distinguishing the extent to which peer choice determines individual choice.

To accurately identify the neighborhood effects concerned in our paper, we take the following measures in the empirical analysis. Firstly, we introduce the sample’s neighbor characteristics and village characteristics into the control variables, which can control the contextual effect and self-selection problem. Secondly, we chose neighborhood children aged 3–6 years old’ and ’Neighborhood serious illness suffering’ as instrumental variables and use the IV-Probit model for regression. Finally, we verify the robustness of the neighborhood effects by using substitution variables, excluding high-income neighbors, constructing false covariate variables, and placebo tests.

### 4.3 Model and variables

Based on the above analysis of the identification strategy of the neighborhood effects, and combined with the data characteristics, the following Probit model is constructed to identify the neighborhood effects of online financial investment in rural places:

$$\text{Probit}\left(ofi_{i}^{C}=1\right)=\varphi \left(\alpha_{0}+\alpha_{1}\;Nofi_{i}^{C}+\alpha_{2}X_{i}+\alpha_{3}Y_{-i}^{C}+\alpha_{4}Z^{C}+ProDummy\right)$$ (1)

Where *ofi*_*i*_^*C*^ is the indicator for the online financial investment of rural households *i* in village *C* (1 = yes; 0 = no), the data is directly derived from a question in the questionnaire—’whether your family purchased any online financial investment products?’.

The core variable is *Nofi*_*i*_^*C*^ (i.e. neighborhood effects), which indicates the average online financial investment within the neighbors of the focal household *i*. The size and significance of the coefficient *α*_*1*_ are the focus of this paper. The measurement of the core explanatory variable must define the scope of ’neighborhood’. Referring to many relevant literature in China, this study labels ’neighborhood’ as all the rural households living in the same administrative village. As mentioned above, the household registration (’Hukou’) system and urbanisation cause few flows in populations, so rural residents rarely choose their neighbors by their preferences. Besides, villagers, particularly in less-developed places, keep a stable and tight social relationship by living together for generations (Liu et al, 2014; Loh&Li, 2013) [55,56]. Thus, we calculate the neighborhood effects using the following equation:

$$Nofi_{i}^{C}=\left(\sum_{i}^{n}\;of\;i_{i}^{C}-of\;i_{i}^{C}\right)/(n-1)$$ (2)

Eq (2) is the measure of neighborhood behavior in our paper. Neighbors’ influence should not contain the effects from one’s own family, so the focal household *i* is excluded. *n* is the number of sample families in the village.

Otherwise, in order to accurately identify the neighborhood effects of online financial investment, we introduce three categories of control variables into the empirical model.

*X*_*i*_ indicates the first kind of control variables, which are a vector of exogenous characteristics of the respondent or his/her family, including gender, age, education, income, assets, liabilities, stock market participation, and consumption expenditure.

$Y_{i}^{C}$ represents the second kind of control variables. They are a vector of neighbors’ background variables. To partially deal with the contextual effect issue, we add neighbors’ age, education, income, assets, and liabilities into basic regression. The calculation method is similar to formula (2), i.e. the average value of all families except the focal family in the same village.

We address the correlated effect issue in various ways, for example, conducting a province-varying fixed effects model and controlling village-based variables (Z^C^), including ’the economic condition’, ’the greening situation of the village’ and ’the aging rate’.

Moreover, we introduce mediating variables by the unary multiple mediation model to explore how neighborhood effects work. The mediating variables include financial knowledge and risk tolerance willingness.

First, we measured financial knowledge using a combination of four questions. The questions are: "Suppose the bank’s annual interest rate is 4%, if you deposit $100 in a bank for a fixed term of 1 year, the principal and interest you will get after 1 year will be?1. less than $104, 2. equal to $104, 3. greater than $104, 4. impossible to calculate "; "Suppose the bank’s interest rate is 5% per year and the inflation rate is 8% per year, what can you buy after a year of saving $100 in the bank that will be? 1. more than a year ago, 2. as much as a year ago, 3. less than a year ago, 4. impossible to calculate"; "Which do you think is riskier in general, equity or debt funds? 1. equity funds, 2. debt funds, 3. never heard of equity funds, 4. never heard of debt funds, 5. neither, 6. the same"; "Which do you think is riskier in general, Main Board stocks or GEM stocks?1. Main Board, 2. GEM, 3. never heard of Main Board stocks, 4. never heard of GEM stocks, 5. Neither heard of them, 6. the same". If the answer to the above question is " impossible to calculate " or " never heard of either", the value is 0. Those questions cover the financial calculating ability, the knowledge about inflation and risk, and financial market knowledge of the sample. The validity and reliability of the resulting scale were then tested based on the responses to these questions. The Cronbach’s alpha of these scales was 0.865, the KMO value was 0.786, and the Bartlett’s test was significant; Besides, the component matrix loadings of each rotation were greater than 0.682, thus the validity and reliability of above scales measuring financial knowledge. Subsequently, financial literacy indicators (*fl*_*i*_^*C*^) were calculated using factor analysis.

Finally, we use the risk tolerance willingness as another mediating, which is the risk a peasant household is willing to take. We choose the following investment choices as the measurement of risk tolerance willingness: "Assume you have some assets to invest in, which type of project would you choose? 1 = Unwilling to take any risk; 2 = Slightly below-average risk, slightly below-average return; 3 = Average risk, average return; 4 = Slightly above-average risk, slightly above-average return; 5 = High risk, high return". Respondents’ responses to this question reflect their risk tolerance, with a larger value indicating higher risk tolerance. This variable is formulated with *riskt*_*i*_. The ratio of each value to the total sample is as follows: The proportion of *riskt*_*i*_ = 1 is 40.8%; The balance of *riskt*_*i*_ = 2 is 25.0%; The proportion of *riskt*_*i*_ = 3 is 21.7%; The samples with *riskt*_*i*_ = 4 accounted for 8.3%; The ratio of *riskt*_*i*_ = 5 is 4.2%.

Even though the above methods have mitigated many identification challenges, there still exists an endogenous threat that stems from the simultaneity (Bertrand et al, 2000; Chen et al., 2008) [57,58]. We implement the IV strategy to eliminate the reflection problem (Angristc, 2014) [59]. As adopted by Gaviria&Raphael (2001) and Ling et al. (2018), [60,61] we select two neighbors’ characteristics as instruments, i.e. ’Neighborhood children aged 3–6 years old’ and ’Neighborhood serious illness suffering’. Children are one of the most vulnerable groups, and more children mean higher risk exposure. On the other hand, more preschool-aged children (usually 3 to 6 years old in China) bring about a high education expenditure since they have must to attend a kindergarten. Therefore, it will lead to less need for online financial investment. Health risk is the common risk faced by rural households, and medical expenditure is also a big part of the rural household payment. So rural households would increase the payment for medical treatment and the liquidity holding for future health risks if there a family member suffers serious illness. That will squeeze out financial asset allocation, which may decrease the likelihood of purchasing online investment products. Thereby, these two instrumental variables may have a strong influence on the neighbors’ online investment products demand, but not a direct impact on the individuals’ propensity to online investment. Besides, in rural China, the birth of children is less susceptible to human intervention because the fertility behavior of young couples is usually natural after marriage and seldom controls the timing of childbirth. So it is to the health condition of family members. Theoretically, the requirements of IVs are satisfied.

More descriptions of the variables are shown in Table 2.

**Table 2:** Variables description.

category	Variables	abbrev	items
Dependent variable	Online financial investment	*ofi*_*i*_^*C*^	Whether your family purchased any online financial investment products? Dummy (1 = yes; 0 = no)
Explanatory variable	Neighborhood online financial investment	*Nofi*_*-i*_^*C*^	Average online financial investment in neighbors’ households. (range: 0–1)
Instrumental variables	Neighborhood children aged 3–6 years old	*Nchild*_*-i*_^*C*^	The average number of children aged 3 to 6 years old in neighbors’ households. Number
	Neighborhood serious illness suffering	*Nillness*_*-i*_^*C*^	The proportion of neighbors whose family members have suffered a serious illness
Mediators	Financial knowledge	*fl*_*i*_^*C*^	Calculated by factor analysis based on 4 financial knowledge questions (index)
	Risk tolerance willingness	*riskt*_*i*_^*C*^	Assume you have some assets to invest in, which type of project would you choose? (1 = Unwilling to take any risk;2 = Slightly below-average risk, slightly below-average return;3 = Average risk, average return;4 = Slightly above-average risk, slightly above-average return;5 = High Risk, High Return)
Rural household characteristics	Gender	*sex*_*i*_^*C*^	Female = 0, male = 1
	Household head age	*age*_*i*_^*C*^	Age (Years)
	Education	*edu*_*i*_^*C*^	Number of years schooling (Years)
	Household income	*income*_*i*_^*C*^	Annual household income. Number (10,000 yuan)
	Total assets	*asset*_*i*_^*C*^	The value of your household assets (10,000 yuan)
	Total debt	*debt*_*i*_^*C*^	The value of your household debt (10,000 yuan)
	Stock purchase	*stock*_*i*_^*C*^	Whether your family purchased any stock? Dummy (1 = yes; 0 = no)
	Household consumption	*consumption*_*i*_^*C*^	Annual household consumption expenditure. Number (10,000 yuan)
Neighborhood characteristics	Neighborhood age	*Nage*_*-i*_^*C*^	Neighborhood age (Years)
	Neighborhood education	*Nedu*_*-i*_^*C*^	The average number of years schooling of the neighboring respondents (Years)
	Neighborhood income	*Nincome*_*-i*_^*C*^	The average number of household income of the neighbors’ families(10,000 yuan)
	Neighborhood assets	*Nasset*_*-i*_^*C*^	The average number of household assets of the neighbors’ families(10,000 yuan)
	Neighborhood debt	*Ndebt*_*-i*_^*C*^	The average number of household debt of the neighbors’ families(10,000 yuan)
Village characteristics	The economic condition	*econd* ^*C*^	How is the village economy developing? (range from1-10;1 is the worst;10 is the best)
	The greening situation	*green* ^*C*^	How green is the village? (range from1-10;1 is the worst;10 is the best)
	The aging rate	*oldrate* ^*C*^	The proportion of the elderly over the age of 70 in the village. (%)

### 4.4 Descriptive statistics

Statistical description results of relevant variables used in this empirical study are shown in Table 3 below:

**Table 3:** Descriptive statistical results.

Variable	Mean	Standard	Minimum	Maximum	Obs
*ofi*_*i*_^*C*^	0.19	0.38	0	1	7165
*Nofi*_*-i*_^*C*^	0.19	0.14	0	1	7165
*Nchild*_*-i*_^*C*^	1.56	0.47	0	3	7165
*Nillness*_*-i*_^*C*^	0.15	0.59	0	1	7165
*fl*_*i*_^*C*^	0.01	0.86	-1.168	1.478	7165
*riskt*_*i*_^*C*^	2.11	2.12	1	5	7165
*sex*_*i*_^*C*^	0.48	0.50	0	1	7165
*age*_*i*_^*C*^	48.46	14.1	20	75	7165
*edu*_*i*_^*C*^	10.88	3.91	0	22	7165
*income*_*i*_^*C*^	4.15	10.45	0.10	48.81	7165
*asset*_*i*_^*C*^	50.28	207.90	2.10	897.30	7165
*debt*_*i*_^*C*^	8.96	59.36	0	406	7165
*stock*_*i*_^*C*^	0.11	0.31	0	1	7165
*consumption*_*i*_^*C*^	11.75	12.29	1.64	174.8	7165
*Nage*_*-i*_^*C*^	37.47	3.92	18.16	48.75	7165
*Nedu*_*-i*_^*C*^	3.25	1.23	1.47	16	7165
*Nincome*_*-i*_^*C*^	4.18	2.03	0.57	12.03	7165
*Nasset*_*-i*_^*C*^	50.31	42.43	8.71	201.10	7165
*Ndebt*_*-i*_^*C*^	8.78	5.81	0	94.18	7165
*econd* ^*C*^	5.31	0.28	1	10	7165
*green* ^*C*^	3.94	1.89	1	10	7165
*oldrate* ^*C*^	0.37	0.10	0	0.656	7165

## 5.Empirical results

### 5.1 Baseline regression result

Our study begins by estimating primary specifications and examining neighborhood effects on online financial investment. Table 4 presents the empirical results, including Probit model estimates in column 1, fixed effect (FE) estimates in column 3, and instrumental variables (IV) estimates in column 5. To facilitate our comparison of the size of coefficients, Table 4 exhibits the marginal effects of the variables. Similarly, the coefficients that appear in the later article are also marginal effects. All specifications include the controls for family characteristics, neighborhood characteristics, and village characteristics.

**Table 4:** Neighborhood effects on online financial investment.

***Panel A***	(1)		(2)			(3)	
Variables	Model 1:Probit		Model 2:FE			Model 3:IV-Probit	
	Coef.	*t-value*	Coef.	*t-value*		Coef.	*t-value*
*Nofi*_*-i*_^*C*^	0.248***	4.101	0.236***	3.358		0.303***	4.508
*sex*_*i*_^*C*^	-0.008	-1.242	-0.008	-1.227		-0.006***	-3.34
*age*_*i*_^*C*^	-0.002***	-7.300	-0.002***	-7.247		-0.001***	-4.160
*edu*_*i*_^*C*^	0.003***	3.101	0.003***	3.318		0.002***	5.146
*income*_*i*_^*C*^	0.002***	5.837	0.002***	5.728		0.001***	7.720
*asset*_*i*_^*C*^	0.001***	2.816	0.001***	2.677		0.001***	3.510
*debt*_*i*_^*C*^	-0.014**	-2.325	-0.011**	-2.272		-0.009**	2.434
*stock*_*i*_^*C*^	0.086***	8.365	0.084***	8.189		0.059	0.640
*consumption*_*i*_^*C*^	0.002	1.165	0.001	1.240		0.001	1.010
*Nage*_*-i*_^*C*^	0.003*	1.736	0.002*	1.850		0.003*	1.688
*Nedu*_*-i*_^*C*^	-0.004	-0.695	-0.005	-0.745		-0.044	-3.730
*Nincome*_*-i*_^*C*^	-0.004*	-1.810	-0.007*	-1.751		-0.006*	-1.702
*Nasset*_*-i*_^*C*^	-0.001*	-1.835	-0.001**	-2.448		-0.001*	-1.680
*Ndebt*_*-i*_^*C*^	0.002	0.444	0.002	0.621		0.002	0.910
*econd* ^*C*^	-0.008	-0.660	-0.0102	-0.698		0.014	0.650
*green* ^*C*^	-0.006	-0.874	-0.006	-0.756		-0.004	-0.67
*oldrate* ^*C*^	0.049	0.975	0.033	0.621		0.043	0.590
*Pseudo R^2*	0.113		0.116			0.136	
*Obs*	7165		7165			7165	
***Panel B***: *First-stage estimation results*							
*Nchild*_*-i*_^*C*^	-0.102***				4.438		
*Nillness*_*-i*_^*C*^	-0.011***				2.432		
*First-stage F value-Weak identification test*	84.32						
*DWH p-Value -Endogeneity test*	0.001						
*Hansen J p-Value-Over-identification test*	0.612						

Note: ***, **, and * refers to p < 0.01, p < 0.05, and p < 0.1, respectively. The ’Coef.’ reported in Panel A are the marginal effects (dy/dx) of the variables.

Firstly, in models 1 and 2, we can see that the coefficients of the neighborhood effects (*Nofi*_*-i*_^*C*^) both are positive and significant at the 1% level as expected. The results show that the direction and significance of the neighborhood effects do not change much, from 0.248 to 0.236, which means that the rural household has an increasing likelihood of online financial investment of 0.236 percentage points as a result of one percentage point increase in the neighbors’ participation rate. The estimation results of models 1 and 2 initially confirm that the average online financial investment in a village has a significant positive impact on online investment. Thus, the neighborhood effects exist in online financial investment.

Our preferred specification is model 3 (IV method). As Manski (1993) argues [62], Probit and FE estimation cannot avoid the endogenous problem caused by simultaneity. To further solve the endogenous problem, we use the IV approach by taking the ’neighborhood children aged 3–6 years old’ and ’neighborhood serious illness suffering’ as the instrumental variables of the potential endogenous regressor. To obtain the marginal effect of the IV-Probit model, we use the maximum likelihood estimation (MLE). The results are shown in model 3 in Table 4. The hypothesis 1 is proved.

As discussed above, these two neighbors’ features are likely to meet the requirements of instrumental variables. This study also runs a series of tests to verify the validity of the instrumental variables. Since the MLE cannot test the weak instruments, instead, we report the first-stage estimates obtained by the two-step method (2SLS) to exclude the possibility of the weak IVs in Panel B. As shown in Table 4, two IVs yield an F-statistics over threshold value 10 (84.32) with a p-value of less than 1% (0.000), which indicates that the weak-instrument issue is not a severe problem in our estimation. The Hansen J p-value of the over-identification test is 0.6120, higher than 0.1, which proves that we do not reject the joint null hypothesis, that is, the over-identification restriction is satisfied. Furthermore, the p-value of the DWH test is 0.001, which rejects the null hypothesis that neighborhood online financial investment is an exogenous variable, implying the existence of the endogeneity problem. Therefore, we are safe to adopt *Nchild*_*-i*_^*C*^ (Neighborhood children aged 3–6 years old) and *Nillness*_*-i*_^*C*^ (Neighborhood serious illness suffering) as the instruments for neighborhood effects.

Column 5 in Table 4 reports the IV estimates. The result implies that a one percentage point increase in the reference group’s purchase leads to a 0.303 percentage point increase in a family’s probability of online investment (significant at 99% confidence). Compared to models 1 and 2, the magnitude of the neighborhood effects estimate has a marked increase. The IV-Probit estimate suggests that the previous estimations are biased; More specifically, the results of Probit and FE both underestimate the neighborhood effects. In summary, this evidence is consistent with the hypothesis that changes in neighbors’ online financial investment behaviors would affect the focal household’s investment behavior. One possible reason is that they assert their neighbors have more private information and therefore make decisions with more value and reference. It is also possible that the rural residents are afraid to become ’special’ under the cultural background of the ’Doctrine of the Mean’ in China, so they are inclined to be in line with their neighbors.

The first-stage estimation results show that both the first IV ’neighborhood children aged 3–6 years old’ and ’Neighborhood serious illness suffering’ negatively affects neighbors’ online financial investment behavior. As mentioned before, the children aged 3 to 6 years begin to receive a preschool education in kindergarten, which raises the education expenditure in a family. Besides, more children bring higher living costs regarding nutrition and health, which may also squeeze the invest budget at a given income. Medical expenditure is the necessary expenditure of farmers, accounting for a large part of the total expenditure. If suffering from serious illness, the neighborhood will increase the payment for medical treatment and hold more liquidity to deal with the health risk in future. Thus, households in rural areas will allocate less money to the online financial investment.

### 5.2 Robustness tests

In this section, we report different robust tests in Table 5 to further investigate the stability of the results in section 5.1. The following robustness checks confirm the existence of the neighborhood effects on decisions concerning online investment products purchase.

**Table 5:** Robustness checks of neighborhood effect.

Variables	Robustness		Robustness		Robustness		Robustness	
	check 1		check 2		check 3		check 4	
	Coef.	t-value	Coef.	t-value	Coef.	t-value	Coef.	t-value
*Nofi*_*-i*_^*C*^	0.295***	4.101	0.252	1.358	0.152	1.002	0.289***	3.102
*Instumental variables*	Yes		Yes		Yes		Yes	
*Household characteristic*	Yes		Yes		Yes		Yes	
*Neighborhood characteristics*	Yes		Yes		Yes		Yes	
*Village characteristics*	Yes		Yes		Yes		Yes	
*Provincial dummies*	Yes		Yes		Yes		Yes	
*Pseudo R^2/ R^2*	0.131		0.120		0.115		0.128	
*Observations*	4777		7165		7165		7165	

Note: ***, **, and * refers to p < 0.01, p < 0.05, and p < 0.1, respectively. The ’Coef.’ reported are the marginal effects (dy/dx) of the variables.

The first concern is that we use the average of the remaining households in a group as the proxy variable when calculating the ’neighborhood effects’. Considering a household’s online financial investment consumption may be affected by specific neighbors with similar annual income rather than by the mean of all the group (Ling et al, 2018) [61], we remove the sample families with the top 1/3 high income in the village and only keep families with similar income levels. The result in column 1 shows that the neighborhood effects still significantly exist.

Then, we run a placebo test for the concern that people make similar purchasing decisions owing to their similar traits, such as income level. First, based on four household characteristics, including the income (continuous variable; unit: yuan), risk attitude (index; range: 0–1), labour (continuous variable), and self-reported social status (1 = lower than average,2 = medium,3 = higher than average), we calculate a Mahalanobis metric *Mm*^*c*^_*i*_ for each household *i* in village *v* of county *c* as Eq (3). Then, we use the nearest-neighbor matching approach to find the exchanged household *j* for household *i*, and *j* must satisfy two requirements: live in the same county but different villages with household *i*; and the nearest Mahalanobis metric to *i*, that is, the absolute value of the difference between two households’ Mahalanobis metrics is the smallest according to Eq (4).

$${\text{Mm}}_{\text{i}}^{\text{C}}=\sqrt{{\left(x_{i}^{d}\right)}^{'\wedge^{-1}}x_{i}^{d}}$$ (3)

$$x_{i}^{d}={\text{x}}_{\text{i}1}-\bar{{\text{x}}_{1}},{\text{x}}_{\text{i}2}-\bar{{\text{x}}_{2}},{\text{x}}_{\text{i}3}-\bar{{\text{x}}_{3}},{\text{x}}_{\text{i}4}-\bar{{\text{x}}_{4}}4\times 1,\;\text{and}\;x^{d}=\left[x_{1}^{d},x_{2}^{d},x_{3}^{d},\ldots ,x_{n}^{d}\right]$$

$$\sum 4\times 4=x^{d}{\left(x^{d}\right)}^{\prime }$$

$$j_{-\wedge }^{C}=min_{j\in N_{-V}^{c}}Mm_{j}^{c}-Mm_{i}^{c}$$ (4)

where *x*_*i*_^*d*^ is a demean column vector of household *i* characteristics. ∑ is the sample covariance matrix. Household *j*_*-v*_^*c*^ is the family with the shortest metric (most similar) from household *i* in the same county but a different village. *N*_*v*_^*c*^ is households who live in county *C* but not in village *v*. The regressions were then conducted using Household *i*’s neighborhood variables and Household *j*_*-v*_^*c*^’s online financial purchases. If the coefficient is significant, it indicates that the positive correlation in baseline regression results from the same background rather than a neighborhood effects. If it is not significant, then the consistency is due to neighborhood effects. The result is listed in column 2 of Table 5. We find that the coefficient of Household *i*’s neighborhood variables is non-significant, which confirm the baseline regression.

In addition, instead of regarding the other village members as a neighborhood group, we assign household *j*’s neighbors as household *i*’s ’fake neighbors’, while assigning *j*’s neighbors to *i*. It is expected that there are no neighborhood effects between a rural household *i* and its fake neighbors because they are not likely to have many opportunities to affect each other due to the long distance between villages. If the fake ’neighborhood online financial investment’ variable is significant, we can consider that similar online financial investment choices may stem from common household characteristics (correlated effect), rather than the truly ’neighborhood effects’. As expected, the estimate in column 3 of Table 5 shows that neighborhood online financial investment is not significant, which indicates the possible ’correlated effect’ may not be a severe problem.

As a final check, we modify the basic model from the Probit model to the ordinary least squares (OLS) model. The neighborhood effects is still significantly positive in the linear OLS regression (in column 4 of Table 5), which further supports the robustness of our finding.

### 5.3 Heterogeneity analysis

It is interesting to separate the samples into different groups to explore possible differences in their susceptibility to peer influence. This part conducts a heterogeneity analysis on gender, age, income, and education level.

## (1) Gender and age

In this part, we divide the total sample into the male and female groups aiming to explore whether the strength of the neighborhood effects on peasant households’ participation in online financial investment varies with the gender. Then regression is conducted respectively using the baseline model. The regression results are shown in columns (1) and (2) of Table 6 below. The coefficients of *Nofi*_*-i*_^*C*^ in the two regression groups are significantly positive, the female group is larger than the male group. The sex*Nofi_*-i*_^*C*^ in column (3) of Table 6 is significantly negative. Accordingly, the neighborhood effects exist in the online financial investment of the male or female group. Specifically, compared with males, most women have limited ways to get information and a lower level of knowledge and communicate more frequently with their friends in their social nets.

**Table 6:** Gender and age.

*Variables*	(1)	(2)	(3)	(4)	(6)	(7)
	*ofi*_*i*_^*C*^	*ofi*_*i*_^*C*^	*ofi*_*i*_^*C*^	*ofi*_*i*_^*C*^	*ofi*_*i*_^*C*^	*ofi*_*i*_^*C*^
	Female	Male	All	Young	Old	All
*Nofi*_*-i*_^*C*^	0.352***	0.201***	0.346***	0.340***	0.212***	0.849***
	(9.030)	(8.061)	(6.189)	(10.076)	(9.069)	(3.165)
sex*Nofi_*-i*_^*C*^			-0.002***			
			(-5.620)			
age*Nofi_*-i*_^*C*^						-0.001***
						(4.751)
*Instrumental variables*	Yes	Yes	Yes	Yes	Yes	Yes
*Household characteristic*	Yes	Yes	Yes	Yes	Yes	Yes
*Neighborhood characteristics*	Yes	Yes	Yes	Yes	Yes	Yes
*Village characteristics*	Yes	Yes	Yes	Yes	Yes	Yes
*provincial dummies*	Yes	Yes	Yes	Yes	Yes	Yes
*Pseudo R^2*	0.161	0.150	0.165	0.120	0.125	0.166
*Obs*	3446	3719	7165	3379	3786	7165

Note: ***, **, and * refers to p < 0.01, p < 0.05, and p < 0.1, respectively. The ’Coef.’ reported are the marginal effects (dy/dx) of the variables.

Besides, the sample is divided into the youth group (aged between 18 and 45) and the elderly group (age greater than 45 years old or above) to verify whether the neighborhood effects differ with age. Then, regression is performed according to the baseline model, and the results are shown in Table 6 below. Columns (4)—(6) indicate that the neighborhood effects exist in all age groups’ online financial investment, where there is a downward trend with the increase of age. In addition, the cross term age*Nofi_*-i*_^*C*^ in column (7) of Table 6 is significantly negative, thus supporting the above conclusion. The difference lies in two factors. One is younger users have stronger social tendencies and frequency. The other is they are more open-minded and have less social experience and knowledge of online finance products, easily being affected by others.

## (2) Income and education

We divide the total sample into high-income and low-income groups according to the average income and then grouped them into regressions. The results are shown in Table 7 (1) and (2). The coefficients of (*Nofi*_*-i*_^*C*^) are significantly positive in both regressions, and income*Nofi_*-i*_^*C*^ in column (3) of Table 7 is significantly positive. This proves that high-income and low-income groups show neighborhood effects in online financial participation, which is more potent for low-income residents. The reasons lie in low-income residents’ lower financial literacy and narrower information channels.

**Table 7:** Income and education.

Variables	(1)	(2)	(3)	(4)	(6)	(7)
	*ofi*_*i*_^*C*^	*ofi*_*i*_^*C*^	*ofi*_*i*_^*C*^	*ofi*_*i*_^*C*^	*ofi*_*i*_^*C*^	*ofi*_*i*_^*C*^
	High income	Low income	All	Low education	High education	All
*Nofi*_*-i*_^*C*^	0.141***	0.351***	0.118***	0.319***	0.252***	0.277***
	(4.131)	(3.177)	(6.233)	(5.040)	(5.164)	(6.172)
income*Nofi_*-i*_^*C*^			0.008***			
			(5.280)			
edu*Nofi_*-i*_^*C*^						-0.003**
						(-2.260)
*Instumental variables*	Yes	Yes	Yes	Yes	Yes	Yes
*Household characteristic*	Yes	Yes	Yes	Yes	Yes	Yes
*Neighborhood characteristics*	Yes	Yes	Yes	Yes	Yes	Yes
*Village characteristics*	Yes	Yes	Yes	Yes	Yes	Yes
*Provincial dummies*	Yes	Yes	Yes	Yes	Yes	Yes
*Pseudo R^2*	0.097	0.157	0.165	0.121	0.080	0.157
*Obs*	4829	2336	7165	4519	2646	7165

Note: ***, **, and * refers to p < 0.01, p < 0.05, and p < 0.1, respectively. The ’Coef.’ reported are the marginal effects (dy/dx) of the variables.

Then, we group the total sample as a low education group (below high school) and a high education group (high school and above) separately regressed. Less-educated residents have insufficient knowledge and the limited ability to gather and analyze information, so they make up by observing other individuals’ financial behaviors. Consequently, as is shown in columns (4)-(6) of Table 7, the coefficients of *Nofi*_*-i*_^*C*^ are all positive while the coefficients of the edu*Nofi_*-i*_^*C*^ are significantly negative. We can conclude that neighborhood effects exist in online financial participation of these two groups mentioned with a downward trend. It may result from the lower education, the more dependent the household would decide.

### 5.4 Mechanism analysis

With the demonstration of neighborhood effects on rural households’ online financial investment, we further investigate the mechanism using a multiple mediation model, in which financial knowledge and risk-taking are mediating variables. To verify the hypotheses above, we construct a multiple mediation model as follows (Preacher,2008;Jing et al.,2022) [63,64]:

$$fl_{i}^{C}=\alpha_{\text{0}}+\alpha_{1}Nofi_{\text{-}i}^{C}+{\sum \alpha_{\text{n}}X}_{i}^{n}+\mu$$ (5)

$$\text{oprobit}\left(riskt_{i}\right)=\eta_{0}+\eta_{1}Nofi_{\text{-}i}^{C}+{\sum \eta_{\text{n}}X}_{i}^{n}+\mu$$ (6)

$$ofi_{i}^{C}=\theta_{\text{0}}+\theta_{\text{1}}Nofi_{\text{-}i}^{C}+\theta_{\text{2}}fl_{i}^{C}+\theta_{\text{3}}riskt_{i}^{C}+\theta_{\text{4}}X_{i}+\theta_{\text{5}}Y_{-i}^{C}+\theta_{\text{6}}Z^{C}+\mu$$ (7)

*ofi*_*i*_^*C*^ and *Nofi*_*-i*_^*C*^ indicate the same variables as model (1). *Fl*_*i*_^*C*^ is one of the mediated variables, which measures the household’s financial literacy. The household’s financial literacy value is a composite index calculated by factor analysis. *Riskt*_*i*_^*C*^ is the other mediated variables measuring the level of risk tolerance willingness a household is willing to take. *X*_*i*_、*Y*_*-i*_^*C*^ and Z^C^ are the control variables which is identical with model (5).

As is shown in Table 8, columns (1)–(2) present the regression results of *Nofi*_*-i*_^*C*^ to financial knowledge (*fl*_*i*_^*C*^) and risk tolerance willingness(*riskt*_*i*_^*C*^), and both the coefficients of *Nofi*_*-i*_^*C*^ are all significantly positive. Accordingly, the online financial investment of others in the same village would enrich financial knowledge and enhance risk-taking. Besides, the significantly positive coefficients of *fl*_*i*_^*C*^ and *riskt*_*i*_^*C*^ in column (3) proves that the online financial investment of rural household is positively related to financial knowledge and risk tolerance willingness. Above all, we can infer that the neighborhood effects on online financial investment can work through financial knowledge and risk tolerance willingness. Therefore, research hypotheses 2–3 are confirmed.

**Table 8:** The mechanism analysis of neighborhood effects.

*Variables*	(1)	(2)	(3)
	OProbit	OProbit	Ols
	*fl*_*i*_^*C*^	*riskt*_*i*_^*C*^	*ofi*_*i*_^*C*^
*Nofi*_*-i*_^*C*^	0.123***	0.021***	0.241***
	(4.154)	(3.571)	(6.420)
*fl*_*i*_^*C*^			0.031**
			(1.967)
*riskt*_*i*_^*C*^			0.042***
			(5.390)
*Instumental variables*	Yes	Yes	Yes
*Household characteristic*	Yes	Yes	Yes
*Neighborhood characteristics*	Yes	Yes	Yes
*Village characteristics*	Yes	Yes	Yes
*Provincial dummies*	Yes	Yes	Yes
*Pseudo R^2*	0.148	0.051	0.128
*Obs*	7165	7165	7165

Note: ***, **, and * refers to p < 0.01, p < 0.05, and p < 0.1, respectively. The ’Coef.’ reported are the marginal effects (dy/dx) of the variables.

## 6.Conclusion

In this paper, we identify the role of neighborhood effects in online financial purchase decisions using the data from CHFS in China. We chose rural households as the study sample to eliminate the correlated effect and control the village characteristics. In addition, we control a series of peer characteristics to address the contextual effect issue. The instrumental variables (IV) method is employed to solve the simultaneity problem. The empirical results find that one rural household’s online financial investment possibility will increase by 0.303 units for each additional unit of neighbors’ investment in online financial products. The four robustness tests’ results confirm neighborhood effects in online financial investment. Meanwhile, we have proved that financial knowledge spillover and risk-taking enhancement significantly mediate neighborhood effects on peasant households’ online financial investment. Our study contributes to prior literature by giving empirical insights into the consequences of neighborhood effects on the online financial investment of peasant households, which conduces to a better understanding of the financial decision-making of rural households. The research may provide a practical implication for popularizing new financial products and the optimal design of policy interventions.

Our research has the following implications: (1) Online financial platforms should fully use the neighborhood effects among rural households to improve residents’ acceptance of new online financial products. (2) The financial regulator departments should pay enough attention to the neighborhood effects and neighborhood effects multipliers when they try to regulate the alarming economic issues in rural areas. (3) Financial knowledge and risk tolerance willingness are significant ways neighborhood effects work. Therefore, changing financial knowledge and risk tolerance willingness are effective measures to promote positive financial behaviors and inhibit the contagion of negative economic behavior among rural residents.

Admittedly, this study has several limitations. The virtual channel of regional neighborhood effects work is a fascinating yet challenging question. Due to data availability, we failed to study further the other mechanisms of how the neighborhood effects work, especially the market sentiment contagion. Other mediators should be considered in future research. Besides, neighborhood effects always work through social networks, so it is crucial to identify the critical points of these networks. That will be an essential topic for further study.

## Supporting information

S1 Data (ZIP)
